# Vertebral Bone Marrow Fat Is independently Associated to VAT but Not to SAT: KORA FF4—Whole-Body MR Imaging in a Population-Based Cohort

**DOI:** 10.3390/nu12051527

**Published:** 2020-05-24

**Authors:** Dunja Hasic, Roberto Lorbeer, Robert C. Bertheau, Jürgen Machann, Susanne Rospleszcz, Johanna Nattenmüller, Wolfgang Rathmann, Annette Peters, Fabian Bamberg, Christopher L. Schlett

**Affiliations:** 1 Department of Diagnostic and Interventional Radiology, Medical Center - Faculty of Medicine, University of Freiburg, 79085 Freiburg, Germany; dunja.hasic@uniklinik-freiburg.de (D.H.); fabian.bamberg@uniklinik-freiburg.de (F.B.); 2 Department of Radiology, Ludwig-Maximilian-University Hospital, 80539 Munich, Germany; roberto.lorbeer@med.uni-muenchen.de; 3 German Center for Cardiovascular Disease Research (DZHK e.V.), 20251 Munich, Germany; peters@helmholtz-muenchen.de; 4 Department of Radiology, Diagnostic and Interventional Radiology, University of Heidelberg, 69117 Heidelberg, Germany; Robert.Bertheau@med.uni-heidelberg.de (R.C.B.); johanna.nattenmueller@med.uni-heidelberg.de (J.N.); 5 Institute for Diabetes Research and Metabolic Diseases (IDM) of the Helmholtz Center Munich, University of Tuebingen, 72072 Tuebingen, Germany; juergen.machann@med.uni-tuebingen.de; 6 German Center for Diabetes Research (DZD), 85764 Neuherberg, Germany; 7 Department of Diagnostic and Interventional Radiology, Section on Experimental Radiology, University Hospital Tuebingen, 72074 Tuebingen, Germany; 8 Institute of Epidemiology, Helmholtz Zentrum München, German Research Center for Environmental Health (GmbH), 85764 Neuherberg, Germany; susanne.rospleszcz@helmholtz-muenchen.de; 9 Department of Biometry and Epidemiology, German Diabetes Center, 40225 Düsseldorf, Germany; rathmann@ddfi.uni-duesseldorf.de; 10 Institute for Cardiovascular Prevention, Ludwig-Maximilian-University-Hospital, 80539 Munich, Germany; 11 Department of Radiology, University Heart Center, University of Freiburg, 79085 Freiburg, Germany

**Keywords:** magnetic resonance imaging, bone marrow, visceral adipose tissue, subcutaneous adipose tissue, metabolic disease

## Abstract

The objective of the current study was to assess the relationship of bone marrow adipose tissue (BMAT) content to abdominal fat depots, including visceral adipose tissue (VAT) and subcutaneous adipose tissue (SAT), as well as cardiovascular risk factors (CVRF) beyond physical activity in a population-based cohort study undergoing whole-body magnetic resonance (MR) imaging. Subjects of the Cooperative Health Research in the Augsburg Region (KORA) FF4 study without known cardiovascular disease underwent fat fraction quantification in vertebrae (BMAT_L1/L2_) via a 2-point T1-weighted volumetric interpolated breath-hold examination (VIBE) Dixon sequence. The same MR sequence was applied to quantify VAT and SAT volume. Subjects’ characteristics, including physical activity, were determined through standardized exams and self-assessment questionnaires. Univariate and multivariate linear regression were applied. In the cohort of 378 subjects (56 ± 9.1years; 42.1% female), BMAT_L1/L2_ was 54.3 ± 10.1%, VAT was 4.54 ± 2.71 L, and SAT was 8.10 ± 3.68 L. VAT differed significantly across BMAT_L1/L2_ tertiles (3.60 ± 2.76 vs. 4.92 ± 2.66 vs. 5.11 ± 2.48; *p* < 0.001), there was no significant differences for SAT (*p* = 0.39). In the fully adjusted model, VAT remained positively associated with BMAT_L1/L2_ (β = 0.53, *p* = 0.03). Furthermore, BMAT_L1/L2_ was associated with age (β = 5.40 per 10-years, *p* < 0.001), hemoglobin A1c (HbA1c; β = 1.55 per 1%, *p* = 0.04), lipids (β = 0.20 per 10 mg/dL triglycerides; β = 0.40 per 10 mg/dL low-density lipoprotein (LDL); β =−3.21 lipid-lowering medication; all *p* < 0.05), and less physical activity (β = 3.7 “no or nearly no exercise” as compared to “≥2 h per week, regularly”, *p* = 0.003); gender was not significantly different (*p* = 0.57). In the population-based cohort, VAT but not SAT were associated with higher BMAT_L1/L2_ independently of physical activity and other cardiovascular risk factors. Further, BMAT_L1/L2_ increased with older age, less physical activity, higher HbA1c, and increased lipids but decreased with lipid-lowering medication.

## 1. Introduction

Cardiovascular disease continues to be the leading cause of morbidity and mortality in both developing and developed countries [1]. Excess body weight is a significant risk factor for cardiovascular disease, and the prevalence of obesity has continued to increase over the past three decades in the western world [2]. Obesity has been classically defined by body mass index (BMI), which is an indicator of general adiposity; however, it does not differentiate between different fat depots. Several studies show that different local fat compartments account for different metabolic effects and different risks for cardiovascular disease [3,4]. The effects of the different fat compartments and their relationship to each other have yet to be fully understood. 

Visceral abdominal adipose tissue (VAT) and subcutaneous abdominal adipose tissue (SAT) have been studied in the past. Overall, VAT has demonstrated a stronger association to cardiovascular risk factors, presence of disease, and the development of major cardiovascular events compared to SAT [5,6]. VAT and SAT are often measured using imaging; different imaging modalities are available for measuring local fat compartments including ultrasound, computed tomography, or magnetic resonance imaging (MRI), but more frequently MRI is used, particularly in epidemiological cohort studies given the low side effects of MRI [7,8,9]. 

Using whole-body MRI, other fat compartments can be assessed beyond VAT and SAT. We recently described a method to measure bone marrow adipose tissue (BMAT) in whole-body MRI [10] and demonstrated that vertebral BMAT is dependent on physical activity (BMAT increases with more physical activity). In contrast, BMAT in the femoral head is not related to physical activity [10]. BMAT, which is composed of bone marrow adipocytes that not only act as storage cells, but also as influencers of our metabolism secreting adipokines, like leptin and adiponectin [11,12,13]. As described above, VAT is also a metabolically active fat compartment shown to secrete vasoactive substances and growth factors [3]. However, the relationship of BMAT with VAT and SAT, as well as with cardiovascular risk factors, is less understood. 

The primary objective of this study was to investigate subjects without cardiovascular disease drawn from a general population that had undergone whole-body MRI and analyze the relationship between BMAT and other fat compartments such as VAT and SAT. Furthermore, analyzing whether these associations were independent of physical activity as well as of cardiovascular risk factors. Finally, we aimed to determine whether gender is an effect modifier for the association between BMAT and VAT given the extensive evidence of different fat distribution across gender.

## 2. Materials and Methods

### 2.1. Study Design and Population

The population was recruited from the prospective cohort study “Cooperative Health and Research in the Region of Augsburg” (KORA), in which 400 subjects underwent whole-body magnetic resonance (MR) imaging as part of the FF4 follow-up [14,15]. Only subjects without a history of cardiovascular disease, defined as validated/self-reported stroke, myocardial infarction, or revascularization, were included. From this analysis, subjects with any possibility of bone marrow cancer were excluded. Further exclusion criteria consisted of a status post-implantation of non-MRI supported devices, cerebral aneurysm clips, and serum creatinine of ≥1.3 mg/dl. The institutional review board of the medical faculty of Ludwig-Maximilian University Munich approved the study. All participants provided written informed consent. 

### 2.2. Magnetic Resonance Imaging

A 3 Tesla Magnetom Skyra (Siemens Healthineers AG, Erlangen, Germany) was used for the whole-body imaging, as described previously [15], which was performed within three months after the first study center visit. As part of the whole-body MR imaging protocol, a coronal 2-point Dixon T1-weighted volumetric interpolated breath-hold examination (VIBE) sequence (repetition time 4.06 ms; echo times 1.26 ms, 2.49 ms; flip angle 4°; slice thickness 1.7 mm) covering the torso was acquired. 

### 2.3. MR Image Analysis: Bone Marrow Adipose Tissue (BMAT) 

DIXON-based water and fat selective images were used to determine BMAT, and image analysis was performed using dedicated software (OsiriX 7.0, Pixmeo SARL, Bernex, Switzerland). The fat content of the bone was determined within a region of interest by the equation below as previously described [10].

$$estimate\;of\;BMAT\;fat\;fraction\;\left(in\;\%\right)=\frac{{mean\;intensity}_{fat\;image}}{{mean\;intensity}_{fat\;image}+{mean\;intensity}_{water\;image}}$$

**Figure 1:**
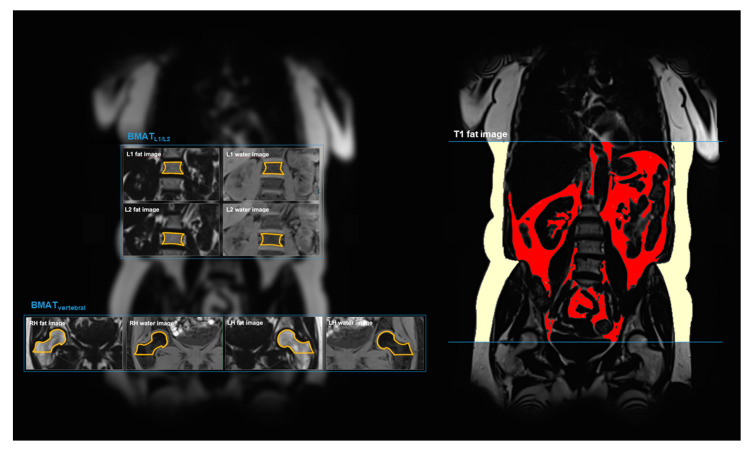
Illustration of the fat measurements in 3 Tesla whole-body magnetic resonance imaging (MRI). For all derived fat depots, a coronal 2-point Dixon T1-weighted sequence was used. The left panel shows the measurements of vertebral and femoral bone marrow adipose tissue content (BMATL1/L2 and BMATfemoral, respectively) by placing a region of interest in the fat and water image and calculating a ratio. The right panel shows the segmentation of subcutaneous adipose tissue (SAT; light yellow) and visceral adipose tissue (VAT; red) of an a 61-year-old male (body mass index (BMI) 29.7 kg/m²).

A similar approach was described in previous studies [10,16,17,18]. For vertebral BMAT, a single coronal image was used to measure BMAT at L1 and L2 of the anterior–posterior diameter of the vertebral body (Figure 1). L1 and L2 were measured separately and averaged afterward. The bone marrow measurements included cancellous bone, which was manually delineated on the fat image and finally copied to the water image. Cortical bone was excluded from the measurements, and mean intensity values were derived. For BMAT at the right and left proximal femur, a single coronal slice was selected, which covered the largest area of the femoral neck (Figure 1). Again, all cancellous bone, but no cortical bone, was included. The right and the left site were measured separately and then averaged.

### 2.4. MR Image Analysis: Visceral and Subcutaneous Adipose Tissue (VAT and SAT)

The amount of VAT and SAT was quantified from axially reconstructed fat selective images (slice thickness 5 mm). A fuzzy-clustering algorithm was applied extracting VAT from the total adipose tissue matrix by using a three-dimensional statistical shape model [19]. VAT and SAT volumes were quantified from femoral heads to the thoracic diaphragm, as shown in an original coronal cross-section in Figure 1 of a 61-year-old male subject. Automatic segmentation took about 2 min per dataset.

### 2.5. Demographics and Physical Activity

The collection of subjects’ characteristics has been described elsewhere [14,15]. Briefly, all subjects went through an examination circle over a year-long period in 2013, where several anthropometric measurements were taken as well as demographics and risk factors selected. A standardized questionnaire was completed by the subjects to evaluate physical activity. Using the questionnaire, we assessed four categories, as used in Bertheau et al. Exercise: “How often do you exercise/work-out?”: ≥2 h per week, regularly; ~1 h per week, regularly; ~1 h per week, irregularly; no or nearly no workout. Non-exercise Walking: “How long do you usually walk on a work day? (e.g., going for a walk, commuting, shopping)”: <0.25 h; 0.25–0.5 h; 0.5–1 h; >1h. Non-exercise Cycling: “How long do you usually ride a bicycle on a work day? (e.g., commuting, shopping)”: <0.25 h; 0.25–0.5 h; 0.5–1 h; >1 h. Non-Exercise Activity at Work: “How would you describe your job/main work?”: no relevant physical labor; light physical labor; moderate physical labor; heavy physical labor [10].

### 2.6. Statistical Analysis

Characteristics of study participants are presented as means and standard deviations (SD) for continuous variables, and as counts and percentages for categorical variables according to BMAT_L1/L2_ tertiles. Characteristic differences among BMAT_L1/L2_ tertiles were evaluated by one-way ANOVA or chi-square test.

Correlations between abdominal adipose tissue (VAT, SAT) and BMAT_L1/L2_ were expressed by scatter plots and Pearson correlation coefficients. Uni- and multivariable associations of abdominal adipose tissue (VAT, SAT) and other risk factors with BMAT_L1/L2_ were assessed by linear regression models providing β-coefficients with 95% confidence intervals (CI). Effect-modifications by sex were evaluated using dedicated interaction terms, and normal distributions of predicted residuals were tested visually.

Multivariable models were adjusted for age, sex, physical activity (model 2), and additionally for diabetes status, hypertension, triglyceride, low-density lipoprotein (LDL)-cholesterol, and lipid-lowering medication (model 3).

A two-sided *p*-value of <0.05 was considered to indicate statistical significance. All analyses were conducted with Stata 16.1 (Stata Corporation, College Station, TX, USA).

## 3. Results

Of a total of 400 subjects, one subject was excluded because of possible bone marrow cancer by history, and a further 21 subjects were excluded because of artifacts affecting the MR measurements critically. The final cohort consisted of middle-aged (*n* = 378; 56.0 ± 9.1 years), Caucasian subjects with an average BMI of 28.1 kg/m^2^. More male than female subjects were included in the study (58% vs. 42% females). The cohort had an average high-density lipoprotein (HDL) of 61.6 mg/dL and average LDL of 140 mg/dL. Detailed characteristics of the study sample are provided in Table 1. Regarding physical activity, 28.6% performed exercise of 2 h regularly or more per week, 30.4% about 1 h regularly per week, 14.8% about 1 h irregularly per week, and 26.5% reported no or nearly no exercise.

### 3.1. Vertebral BMAT and Its Association to Demographics and Risk Factors

Mean BMAT_L1/L2_ was 54.3 ± 10.1% ranging from 15.7% to 78.2%. Tertiles of BMAT_L1/L2_ were 15.7–50.7%, 50.8–59.1%, and 59.2–78.2%. The association of BMAT_L1/L2_ with physical activity has been previously described [10]. Furthermore, age, glucose tolerance, hemoglobin A1c (HbA1c), hypertension (including antihypertensive medication), triglyceride levels, total cholesterol, LDL, and presence of lipid-lowering medication differed significantly between tertiles of BMAT_L1/L2_ (all *p* ≤ 0.04; Table 1). Subjects in the lower tertile for BMAT_L1/L2_ had lower HbA1c, lower triglycerides, lower LDL, and were younger than the patients in the middle and highest tertiles. Interestingly, BMI did not differ between tertiles of BMAT_L1/L2_ (*p* = 0.22). Furthermore, there was no relationship between BMAT_L1/L2_ and gender (*p* = 0.51; Table 1).

**Figure 2:**
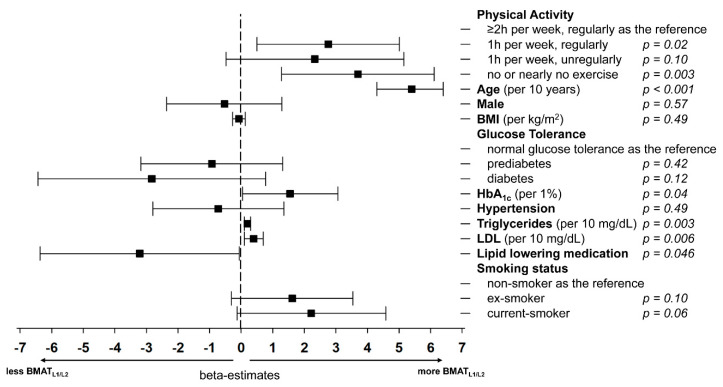
Forrest-plot demonstrating the multivariate association of demographics and risk factors with vertebral bone marrow adipose tissue (BMATL1/L2). BMI denotes body-mass-index; HbA1c, hemoglobin A1c; LDL, low-density lipoprotein.

In multivariate analysis, age, less physical activity, increased HbA1c, and increased lipids remained significantly associated with BMAT_L1/L2_ leading to an increase in fat content (Figure 2), while the lipid-lowering medication is leading to a decrease in fat content.

### 3.2. Relationship of Vertebral BMAT with VAT and SAT

**Figure 3:**
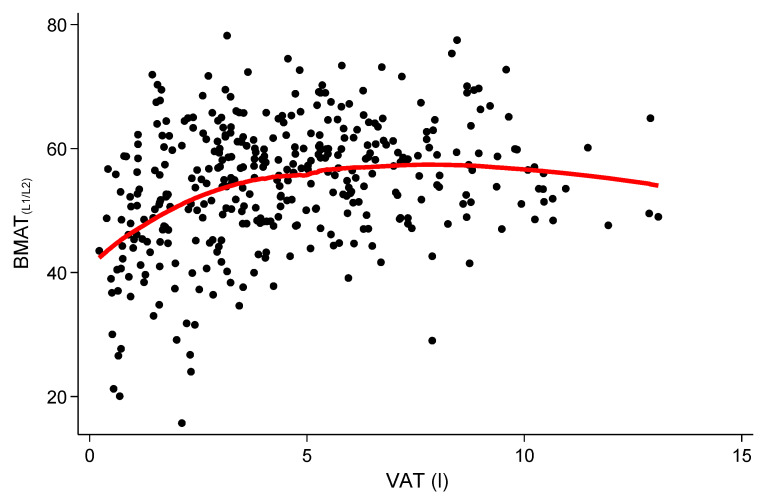
Local-weighted regression illustrated as a fitting curve. A potential saturation function can be argued between vertebral bone marrow adipose tissue (BMATL1/L2) and visceral adipose tissue (VAT); the fitting curve was smoothed.

Mean volumes were 4.54 ± 2.71 L and 8.10 ± 3.68 L for VAT and SAT, respectively. While VAT differed significantly across BMAT_L1/L2_ tertiles (3.60 ± 2.76 vs. 4.92 ± 2.66 vs. 5.11 ± 2.48, respectively; *p* < 0.001), there were no significant differences for SAT (7.82 ± 4.28 vs. 8.45 ± 3.75 vs. 8.04 ± 2.88, respectively; *p* = 0.39). Similar results considered a continuous approach were observed (Figure 2), where BMAT_L1/L2_ was significantly correlated with VAT (*r* = 0.29; *p* < 0.001) but not with SAT (*r* = 0.09; *p* = 0.09). VAT remained positively associated with BMAT_L1/L2_ in a simple model adjusted for age, gender, and physical activity (β = 0.66, *p* = 0.002) and in a fully adjusted model including all potential confounders (β = 0.53, *p* = 0.03; Table 2). As predefined, the univariate associations were also assessed separately for women and men. For both genders, the associations between VAT and BMAT_L1/L2_ were significant (women: β = 1.97, 95% CI: 1.17–2.76, *p* < 0.001 and men: β = 1.15, 95% CI: 0.69–1.61, *p* < 0.001), while the effect was stronger in women than in men (*p* < 0.001 for interaction). Given the correlation distribution between BMAT_L1/L2_ and VAT, local-weighted regressions were performed and are displayed in Figure 3 as a (smoothed) fitting curve illustrating the potential of a saturation function between BMAT_L1/L2_ and VAT.

SAT was associated with BMAT_L1/L2_ in none of the models (all *p* ≥ 0.09; Table 2).

### 3.3. Relationship of Femoral BMAT with VAT and SAT

Mean BMAT_femoral_ was 87.3 ± 6.0% ranging from 47.2% to 95.0%; BMAT was a consistently higher fat content in the femoral than in the vertebrae. A correlation coefficient of 0.46 (*p* < 0.001) was observed between BMAT_L1/L2_ and BMAT_femoral_. Accordingly, BMAT_femoral_ increased across tertiles of BMAT_L1/L2_ (84.6 ± 7.4% vs. 87.7 ± 4.7% vs. 89.9 ± 3.4%, respectively; *p* < 0.001). BMAT_femoral_ itself was not correlated with VAT (*r* = 0.12, *p* = 0.19), but demonstrated a negative correlation with SAT (*r* = −0.22, *p* = 0.002). In the linear regression, BMAT_femoral_ remained inversely associated with SAT independent of potential confounders (β = −0.28, 95% CI: −0.51–−0.05, *p* = 0.02 in the fully adjusted model). 

## 4. Discussion

Our study focused on the association between VAT and SAT when compared to vertebral BMAT content in Caucasian adults drawn from a general population without cardiovascular disease. We demonstrated that increased VAT is positively associated with BMAT_L1/L2_ independent of physical activity as well as other potential confounders. The significant association between VAT and BMAT_L1/L2_ could not be shown with the SAT measurements. We found that there was a correlation between BMAT_L1/L2_ in both sexes with a significant sex interaction for the effect. Assessment of the correlation plots between VAT measured as fat volume, and BMAT_L1/L2_, measured as a fat content, suggests a saturation function. Additionally, BMAT_L1/L2_ increased independently with increasing age, HbA1c, and different lipids, but to a lesser extent when the subjects were under lipid-lowering medication. Overall, our results demonstrate that vertebral BMAT is a significant fat depot involved in the pathway of metabolic-related disorders.

Compared to a previous research study in humans that looked at young healthy adults under the age of 25, no association was found between BMAT and VAT or SAT [20]. These patients showed no increased risk in cardiovascular disease, whereas our older population with an average age of 56 years did. Not only did the patients differ in age but also BMI and fat percentage. The average BMI of the younger patients was 23.8 ± SD kg/m^2^ for the males and 23.6 ± SD kg/m^2^ for the females. In contrast, our average BMI was higher, measuring in at 27.7 kg/m^2^ and demonstrated higher standard deviations. The older population in our study also had higher average VAT (4.54 L) and SAT (8.10 L), the younger cohort had VAT levels under 1 L and SAT levels under 3 L. This further promotes the fact that with increasing age, the effects of adipose tissues become more relevant and more varied. Aging is associated with degenerative processes of bone marrow, characterized by an expansion of adipose tissue at the expense of staminal cells; this could pose a potential relationship between visceral obesity, metabolic syndrome, and age-related aplastic anemia.

The most similar study to our is by Bredella et al. [21], whereby they found a comparable association between vertebral BMAT and VAT, only their primary focus group was obese women (healthy premenopausal women with an average BMI of 29.5 ± 6.9 kg/m^2^ and a younger average age compared to our cohort of 32.8 ± 7.1 years). Two facts must be considered when comparing our results with Bredella et al. [21], first, we demonstrated that the association was strong for both men and women, and second, the strength of the correlation between BMAT and VAT decreased when VAT increased (given the shape of a “saturation function”). Despite the differences of our cohorts, we similarly found that higher levels of vertebral bone marrow fat significantly correlated to increasing levels of VAT, albeit to a different extent. In both studies, the regression analysis of BMAT on VAT showed a positive correlation between BMAT and VAT, which in our study also remained significant in the males after controlling for physical activity, age, gender, and other cardiovascular risk factors. Baum et al. showed comparable vertebral BMAT in diabetic and healthy postmenopausal women [22]. In this study, BMAT was significantly correlated to VAT and HbA1c only in the diabetic group. An intriguing aspect of this observation in a small number of subjects is that BMAT of patients with diabetes is characterized by a different fatty acid composition with a lower amount of unsaturated fat. This suggests that not only the percentage of BMAT but also its composition might influence the metabolic impact of this endocrine organ. BMAT of different skeletal areas is also hypothesized to have different metabolic activities, and different effects on metabolic disease such as diabetes, but the involvement is still unclear. The microenvironment of the different areas would depend on osteoblasts, osteoclasts, hematopoietic, and other cells that regulate bone marrow adipocyte function [23]. Amounts of BMAT show strong relations to other diseases such as osteoporosis. There has been a strong relationship between the amount of BMAT and bone loss emphasizing its potential pathophysiological role in osteoporosis [23]. BMAT differentiation is a characteristic feature of some kinds of osteoporosis, such as glucocorticoid-induced osteoporosis or age-related osteoporosis. This differentiation would make it a strong and reliable indicator of bone integrity for the management of osteoporosis [23]. Studies show that weight reduction can also lead to bone loss, specifically in anorexia nervosa, but also following bariatric surgery [24,25]. These more drastic changes to body composition, causing significant loss of fat tissue, can show that different fat compartments react differently to metabolic changes. The development of bone marrow adipose tissue, when other tissues are being depleted, is not fully understood but seems to depend on the patient’s metabolic status and age [24,25]. In a study performed in 50 subjects applying proton magnetic resonance spectroscopy, Machann et al. revealed lower unsaturation in subjects with higher VAT volume and a correlation between unsaturation in the yellow bone marrow and insulin sensitivity [26]. However, such data are not available for our population as the determination of fatty acid composition requires spectroscopy of advanced multi-echo Dixon techniques. A study by Gilsanz et al. analyzing the reciprocal relations of SAT and VAT to bone structure and strength gave us more insight on the inverse correlation we observed with BMAT and SAT measurements, primarily focusing on the Femur. They found that VAT and SAT have opposite effects on the appendicular skeleton. SAT was shown to relate positively to bone structure and strength, while VAT had the opposite effect and was further classified as a pathogenic fat depot [27].

The current study had some limitations that should be noted. Given the cross-sectional design of our study, we cannot establish any causality in the observed associations. Further, a possible confounder was its design as a case-control study focused on prediabetes and diabetes; nevertheless, the stratification of glucose tolerance did not remain in the multivariate model, potentially due to collinearity with HbA1c, which was a significant predictor for vertebral BMAT. Also, our MRI sequence did not account for T2* effects and did not allow sub-stratification of different lipids [7,28]. However, this analysis looked at relative differences between groups and was not focused on estimating absolute values as carefully as possible.

## 5. Conclusions

In a population-based cohort of subjects without known cardiovascular disease, VAT but not SAT were associated with higher vertebral BMAT levels independent of physical activity and other cardiovascular risk factors. Furthermore, vertebral BMAT was associated with older age, higher HbA1c, and increased lipids. Overall, vertebral BMAT is a relevant fat depot involved in the pathway of metabolic-related disorders.

## Figures and Tables

**Table 1:** Characteristics of the entire cohort study.

	All	Lower Tertile BMAT_L1/L2_	Mid Tertile BMAT_L1/L2_	Higher Tertile BMAT_L1/L2_	*p*-Value
		(15.7–50.7%)	(50.8–59.1%)	(59.2–78.2%)	
*N*	378	126	126	126	
Age (years)	56.0 (±9.1)	50.6 (±7.9)	56.9 (±8.7)	60.6 (±7.6)	<0.001
Female (%)	159 (42.1%)	54 (42.9%)	48 (38.1%)	57 (45.2%)	0.51
BMI (kg/m^2^)	28.1 (±4.8)	27.7 (±5.2)	28.7 (±5.1)	27.9 (±4)	0.22
Glucose tolerance					0.02
Normal glucose tolerance	230 (60.9%)	90 (71.4%)	67 (53.2%)	73 (57.9%)	
Prediabetes	95 (25.1%)	27 (21.4%)	38 (30.2%)	30 (23.8%)	
Diabetes	53 (14.0%)	9 (7.1%)	21 (16.7%)	23 (18.3%)	
HbA1c (%)	5.6 (±0.7)	5.4 (±0.8)	5.7 (±0.7)	5.7 (±0.7)	<0.001
Hypertension	127 (33.6%)	30 (23.8%)	48 (38.1%)	49 (38.9%)	0.02
Systolic RR (mmHg)	121.1 (±16.6)	117.5 (±16.5)	122.2 (±18.0)	123.5 (±14.7)	0.01
Diastolic RR (mmHg)	75.6 (±9.9)	74.3 (±9.8)	76.6 (±10.8)	76.0 (±9.0)	0.16
Antihypertensive medication	93 (24.6%)	22 (17.5%)	32 (25.4%)	39 (31%)	0.04
Triglyceride levels (mg/dL)	132.4 (±85.8)	112.1 (±62.3)	138 (±85.9)	147 (±101.4)	0.003
Total cholesterol (mg/dL)	217.9 (±36.2)	205.7 (±31.6)	220.9 (±36.3)	227.1 (±37.5)	<0.001
HDL (mg/dL)	61.6 (±17.4)	62.0 (±18.4)	60.5 (±17.8)	62.1 (±16.1)	0.72
LDL (mg/dL)	140 (±32.9)	130.4 (±27.8)	143.5 (±35.0)	146 (±33.6)	0.001
Lipid lowering medication	38 (10.1%)	7 (5.6%)	11 (8.7%)	20 (15.9%)	0.02
Smoking status					0.15
Non-smoker	138 (36.5%)	57 (45.2%)	39 (31%)	42 (33.3%)	
Ex-smoker	163 (43.1%)	48 (38.1%)	60 (47.6%)	55 (43.7%)	
Current-smoker	77 (20.4%)	21 (16.7%)	27 (21.4%)	29 (23%)	

BMAT denotes bone marrow adipose tissue; BMI, body-mass-index; HbA1c, hemoglobin A1c; RR, arterial blood pressure; HDL, high-density lipoprotein; LDL, low-density lipoprotein.

**Table 2:** Univariate and multivariate associations of vertebral bone marrow adipose tissue (BMAT_L1/L2_) with visceral adipose tissue (VAT) and subcutaneous adipose tissue (SAT). The simple model included age, sex, and physical activity, whereas the fully adjusted model included age, sex, physical activity, diabetes status, hypertension, triglyceride, LDL-cholesterol, and lipid lowering medication.

Vertebral Bone Marrow Adipose Tissue (BMAT_L1/L2_)		
	β (95% CI)	*p*-Value
Visceral Adipose Tissue (VAT)		
Univariate	1.07 (0.71–1.43)	<0.001
Simple model	0.66 (0.25–1.06)	0.002
Fully adjusted model	0.53 (0.07–1.00)	0.03
Subcutaneous Adipose Tissue (SAT)		
Univariate	0.24 (−0.04–0.52)	0.09
Simple model	0.10 (−0.16–0.35)	0.44
Fully adjusted model	0.03 (−0.24–0.30)	0.80
